# Evaluation of Mannose Binding Lectin Gene Variants in Pediatric Influenza Virus-Related Critical Illness

**DOI:** 10.3389/fimmu.2019.01005

**Published:** 2019-05-08

**Authors:** Emily R. Levy, Wai-Ki Yip, Michael Super, Jill M. Ferdinands, Anushay J. Mistry, Margaret M. Newhams, Yu Zhang, Helen C. Su, Gwenn E. McLaughlin, Anil Sapru, Laura L. Loftis, Scott L. Weiss, Mark W. Hall, Natalie Cvijanovich, Adam Schwarz, Keiko M. Tarquinio, Peter M. Mourani, PALISI PICFLU Investigators, Adrienne G. Randolph

**Affiliations:** ^1^Division of Critical Care Medicine, Department of Anesthesiology, Critical Care and Pain Medicine, Boston Children's Hospital and Department of Anaesthesia, Harvard Medical School, Boston, MA, United States; ^2^Divisions of Pediatric Critical Care and Pediatric Infectious Diseases, Department of Pediatrics, Mayo Clinic, Rochester, MN, United States; ^3^Foundation Medicine Inc., Cambridge, MA, United States; ^4^Wyss Institute at Harvard University, Boston, MA, United States; ^5^Influenza Division, US Centers for Disease Control and Prevention, Atlanta, GA, United States; ^6^Laboratory of Clinical Immunology and Microbiology, National Institute of Allergy and Infectious Diseases, National Institutes of Health, Bethesda, MD, United States; ^7^Division of Pediatric Critical Care Medicine, Department of Pediatrics, University of Miami Miller School of Medicine, Miami, FL, United States; ^8^Critical Care Medicine Division, Department of Pediatrics, Children's Hospital of Los Angeles, University of California, Los Angeles, Los Angeles, CA, United States; ^9^Section of Critical Care Medicine, Department of Pediatrics, Texas Children's Hospital, Houston, TX, United States; ^10^Department of Anesthesiology and Critical Care, Children's Hospital of Philadelphia, Philadelphia, PA, United States; ^11^Division of Critical Care Medicine, Department of Pediatrics, Nationwide Children's Hospital, Columbus, OH, United States; ^12^Department of Pediatrics, Benioff Children's Hospital Oakland, University California San Francisco, Oakland, CA, United States; ^13^Department of Pediatrics, Children's Hospital of Orange County, Orange, CA, United States; ^14^Division of Pediatric Critical Care Medicine, Department of Pediatrics, Children's Healthcare of Atlanta at Egleston, Emory University School of Medicine, Atlanta, GA, United States; ^15^Section of Critical Care Medicine, Department of Pediatrics, University of Colorado School of Medicine and Children's Hospital Colorado, Aurora, CO, United States; ^16^Department of Pediatrics, Harvard Medical School, Boston, MA, United States

**Keywords:** MBL, influenza, pediatric, methicillin-resistant *Staphylococcus aureus*, critical illness, sepsis, mortality

## Abstract

**Background:** Mannose-binding lectin (MBL) is an innate immune protein with strong biologic plausibility for protecting against influenza virus-related sepsis and bacterial co-infection. In an autopsy cohort of 105 influenza-infected young people, carriage of the deleterious MBL gene *MBL2*_Gly54Asp(“B”) mutation was identified in 5 of 8 individuals that died from influenza-methicillin-resistant *Staphylococcus aureus* (MRSA) co-infection. We evaluated *MBL2* variants known to influence MBL levels with pediatric influenza-related critical illness susceptibility and/or severity including with bacterial co-infections.

**Methods:** We enrolled children and adolescents with laboratory-confirmed influenza infection across 38 pediatric intensive care units from November 2008 to June 2016. We sequenced *MBL2* “low-producer” variants rs11003125(“H/L”), rs7096206(“Y/X”), rs1800450_Gly54Asp_(“B”), rs1800451_Gly57Glu_(“C”), rs5030737_Arg52Cys_(“D”) in patients and biologic parents. We measured serum levels and compared complement activity in low-producing homozygotes (“B/B,” “C/C”) to HYA/HYA controls. We used a population control of 1,142 healthy children and also analyzed family trios (PBAT/HBAT) to evaluate disease susceptibility, and nested case-control analyses to evaluate severity.

**Results:** We genotyped 420 patients with confirmed influenza-related sepsis: 159 (38%) had acute lung injury (ALI), 165 (39%) septic shock, and 30 (7%) died. Although bacterial co-infection was diagnosed in 133 patients (32%), only MRSA co-infection (*n* = 33, 8% overall) was associated with death (*p* < 0.0001), present in 11 of 30 children that died (37%). *MBL2* variants predicted serum levels and complement activation as expected. We found no association between influenza-related critical illness susceptibility and *MBL2* variants using family trios (633 biologic parents) or compared to population controls. *MBL2* variants were not associated with admission illness severity, septic shock, ALI, or bacterial co-infection diagnosis. Carriage of low-MBL producing *MBL2* variants was not a risk factor for mortality, but children that died did have higher carriage of one or more B alleles (OR 2.3; *p* = 0.007), including 7 of 11 with influenza MRSA-related death (vs. 2 of 22 survivors: OR 14.5, *p* = 0.0002).

**Conclusions:** *MBL2* variants that decrease MBL levels were not associated with susceptibility to pediatric influenza-related critical illness or with multiple measures of critical illness severity. We confirmed a prior report of higher B allele carriage in a relatively small number of young individuals with influenza-MRSA associated death.

## Introduction

Severe sepsis is the most common cause of death in infants and children across the world (1). Influenza virus is a common global pathogen causing severe sepsis, and annually leads to 100–350 deaths and over 25,000 hospitalizations in North American children (2–4). Influenza suppresses the immune system, allowing respiratory tract colonizers to invade and cause bacterial co-infection, a major contributor to influenza-related morbidity and mortality (5). In 2003, methicillin-resistant *Staphylococcus aureus* (MRSA) emerged in the United States as a major co-infecting bacterial organism in children with influenza virus infection and an independent predictor of death (6). In comparison to healthy adults, children are heavily reliant on innate immunity for protection against influenza virus and bacterial pathogens as they have limited adaptive immunity and fewer years of exposure to develop anti-microbial antibodies (7).

The wide spectrum of influenza virus-related disease severity is likely influenced by host genetics. Novel primary immunodeficiencies to influenza and other common viruses have been identified in children previously thought to be healthy (8–10). Most are in interferon regulatory genes, essential for innate immunity to viruses (11). However, other gene pathways may influence disease severity, particularly in relation to bacterial co-infection. Mannose-binding lectin (MBL), a key innate immunity pattern-recognition protein, activates the lectin complement pathway. MBL has strong biologic plausibility as an innate immunity candidate protein that could protect against influenza-related sepsis with and without bacterial co-infection (12). MBL binds to microbial surface glycosylation residues and targets influenza virus via direct neutralization, by recognition of influenza hemagglutinin surface proteins on infected cells, and can also ameliorate severity by defending against bacterial pathogens (13–15). Additionally, influenza virus uses a glycan-binding entry mechanism to invade host cells, and lectins such as MBL may interfere directly with entry of the pathogen into the cell (16).

**Figure 1:**
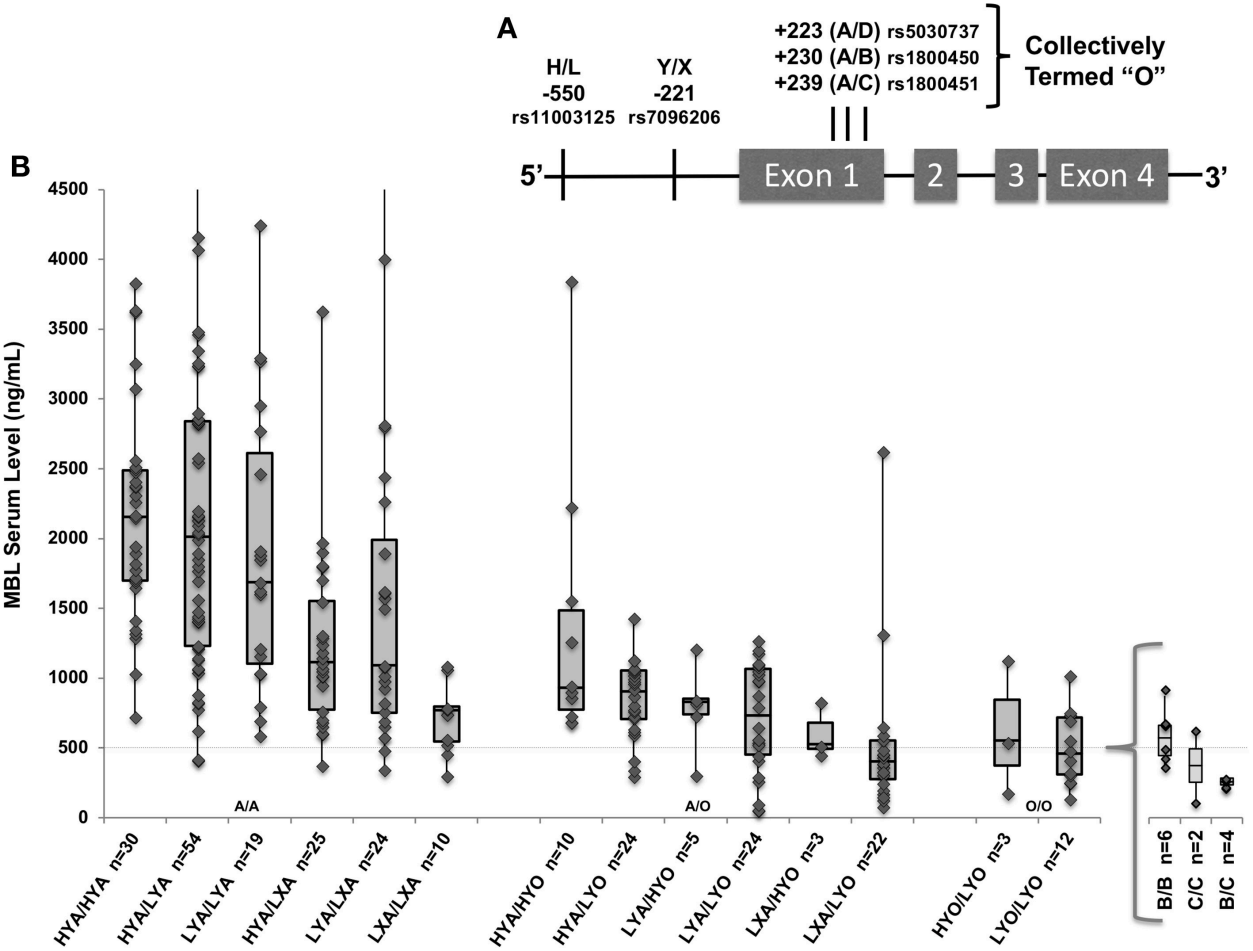
**(A)** *MBL2* gene with known low-producer variant polymorphisms. Marked positions denote promoter variants H/L and Y/X, and exon 1 variants “B,” “C,” and “D” (collectively termed “O”). **(B)** The gene-to-level analysis (*n* = 265) included all genotyped patients who also had measured MBL levels. Box and whisker plots indicate serum level quartiles: 1st quartile (bottom line) 2nd/3rd quartile (box) with median at line, and 4th quartile (top line). *MBL2* haplotypes combined into diplotypes with associated serum MBL levels (*n* = number of patients included); wild type (A/A), heterozygote (A/O), and homozygotes (O/O) with additions of low-producing promoters from left-to-right within each X-axis group. Specific variants within LYO/LYO homozygotes are shown in insert; there were not significant differences in levels among B/B, C/C, and B/C homozygotes. Four patients (3 HYA/LYA, 1 LYA/LXA) had values > the Y axis set maximum of 4,500 ng/mL. Dashed line indicates 500 ng/mL throughout.

MBL serum levels and functional activity are strongly influenced by five single nucleotide polymorphisms (SNPs) in the *MBL2* gene. As shown in Figure 1A, three *MBL2* missense mutations in Exon 1, “B” (rs1800450_A; codon 54 Gly to Asp), “C” (rs1800451_A; codon 57 Gly to Glu), and “D” (rs5030737_T; codon 52 Arg to Cys), combine with *MBL2* promoter polymorphisms “H/L” and “Y/X” to form low-, intermediate- and high-producing MBL haplotypes (17, 18) (see Figure 1B). Although all 3 exon mutations are associated with the lowest levels of MBL, only the B variant Asp residue has been reported to destabilize circulating MBL oligomers having a dominant effect when present, further decreasing functional activity (15, 19, 20). MBL deficiency is common, and may occur in 30% of the population depending on what MBL level cutoff is used for defining it; deficiency has been defined variably as <1,000, <500, <200, or <100 ng/mL (21). In critically ill patients, serum levels may be influenced by inflammation, diluted by fluid resuscitation, or raised via fresh frozen plasma transfusion, so genotype has been used as an inexact proxy to estimate pre-illness MBL deficiency (17, 22).

Many studies have evaluated the association between MBL genotypes and sepsis, with most comparing carriage of low-MBL producing mutations in B, C or D mutation in exon 1 (termed “O”) to wild-type (“A”), but the studies have shown conflicting and inconclusive results (23). A limited number of small studies have evaluated associations between MBL and influenza-related critical illness. Herrera-Ramos *et al* reported no increased frequency of low-producing MBL genotypes in 93 adult Spanish inpatients and outpatients infected with 2009 pandemic H1N1 (H1N1_pdm_09) (24) Higher MBL serum levels were associated with mortality in 27 influenza H1N1_pdm_09-infected critically ill adults (25) whereas 12 critically ill pediatric patients had lower levels compared to ward patients (26). A case series comparing *MBL2* genotypes in 100 fatal pediatric influenza cases (from autopsy samples) with a pediatric population control cohort did not find differences in the frequency of *MBL2* variants known to influence MBL levels after stratifying by ethnicity, but fatal influenza cases with MRSA co-infection were more likely to carry the B mutation (27).

The 2017 World Health Organization Public Health Research Agenda emphasized that identifying host genetic factors influencing influenza susceptibility and severity is paramount for targeting prevention and identifying novel therapeutics (28). MBL repletion is feasible (29) for influenza-infected children predicted to have low or deficient levels. Identification of associations between MBL variants known to influence levels, influenza-related sepsis, and bacterial co-infection could allow opportunities for precision diagnostics and interventions. Therefore, we evaluated associations between *MBL2* variants and overall influenza susceptibility, severity, and bacterial co-infection in a multicenter prospective cohort of critically ill children and adolescents in the Pediatric Intensive Care Influenza (PICFLU) Study.

## Materials and Methods

From November 2008 through June 2016, the PICFLU Study prospectively enrolled patients (<21 years of age) admitted to 38 PICUs in the Pediatric Acute Lung Injury and Sepsis Investigator's (PALISI) Network with suspected or confirmed community-acquired influenza infection. Details of the PICFLU Study design have been previously published (7, 11, 30, 31). Beginning in fall 2010, patients with known risk factors for becoming severely ill with influenza virus such as immunodeficiency, severe chronic lung or heart conditions, were excluded to enrich for identification of genetic susceptibility factors. Testing for influenza virus and other viral pathogens was performed at the enrolling site and in collected respiratory samples using sensitive PCR testing (30, 31). Bacterial co-infection was defined as a diagnosis at the clinical site with microbiologic identification of the pathogen within 72 h prior to or after PICU admission (to exclude hospital-acquired infection). Cultures had to come from a sterile site: endotracheal or bronchoscopic specimen, bloodstream or pleural fluid (31). Patients were defined as previously healthy if they had no underlying comorbidities and were on no chronic medications. Sepsis was defined using the 2005 International Consensus Conference on Pediatric Sepsis criteria (32). Illness severity was assessed by the Pediatric Risk of Mortality (PRISM) III Score (33). The American European Consensus Conference Criteria were used for diagnosis of acute lung injury (ALI) and acute respiratory distress syndrome (ARDS) (34). We collected samples from pediatric patients (blood) and their parents (saliva). Blood was collected as close to admission as possible and used both for DNA extraction and MBL serum level measurements. Each enrolling site received site Institutional Review Board approval. Written informed consent was obtained from a parent or legal guardian for patients and for each parent contributing their own sample for DNA. The population control cohort included healthy adolescents (12–19 years old) from the National Health and Nutrition Examination Survey (NHANES) (35) who had *MBL2* genotyping available (27).

### Serum MBL Evaluation, MBL2 Genotyping, and MBL Functional Assessment

Genomic DNA from peripheral blood was extracted using the Gentra Puregene Blood kit (Qiagen). Saliva samples were collected using Oragene Saliva kits (DNA Genotek Inc., Ontario, Canada). DNA extraction followed manufacturer's recommendations. We used TaqMan assays to genotype samples for single nucleotide polymorphism (SNPs) using the TaqMan OpenArray® SNP Genotyping Platform (Applied Biosystems, Foster City, CA). We genotyped the following SNPs known to influence MBL levels from both patients and biologic parents: rs7096206 (“Y/X”), rs11003125 (“H/L”), rs1800450_A_Gly54Asp_ (allele “B”), rs1800451_A_Gly57Glu_ (allele “C”), and rs5030737_T_Arg52Cys_ (allele “D”) (see Figure 1A).

To identify rare deleterious variants in *MBL2*, we performed targeted re-sequencing of the exons and nearby regions using Illumina TruSeq Custom Amplicon kit (TSCA, Illumina, San Diego, CA) as described previously (30), and this was compared with Taqman genotyping. In samples where genotyping remained inconclusive, Sanger sequencing for the 5 SNPs in *MBL2* was performed.

For patients enrolled prior to May 2014, MBL serum level measurements were done at the Cytokine Reference Laboratory using a commercial enzyme-linked immunosorbent assay kit from R&D Systems (Minneapolis, MN).

MBL protein activity was evaluated by measuring lectin pathway complement fixation in the sera of homozygote patients, either “B/B” (*n* = 4) or “C/C” (*n* = 2), and compared to wild type controls (HYA/HYA; *n* = 6). There were no “D/D” homozygotes for evaluation. Dilutions of patient and control sera were incubated on mannan (Sigma M7504) coated plates in Tris buffered saline with Tween and 5 mM CaCl2 (Boston Bioproducts). After incubation at 37°C and rinsing, deposited C3 fragments were detected with HRP-labeled polyclonal sheep anti-human C3c (BioRad 2222-6604P) and measured by ELISA. Lectin pathway activity was determined by comparing absorbance at 450 nm of C3c bound to mannan from patient sera as previously described (36, 37).

### Statistical Analyses

*MBL2* genotypes were correlated with MBL serum levels to ensure that previously described (17, 38) relationships between *MBL2* variants and serum MBL levels were present in the PICFLU cohort. A multiple linear regression model, adjusting for gender, bacterial co-infection, age, race, and influenza status, was used to evaluate associations between variant alleles and serum levels. Frequencies of allele distribution were also compared between influenza virus subgroups (H1N1_pdm_09 versus non-H1N1_pdm_09; influenza A vs. B) and racial groups (all races, white only, white non-Hispanic). Categorical variables were compared using Chi-square test or Fisher's Exact Tests and continuous variables with the Wilcoxon-Mann-Whitney test.

*MBL2* variant frequencies vary by ethnicity and race. To control for possible confounding by population substructure, we used family-based analyses (PBAT/HBAT) (39–41) based on the Transmission Disequilibrium Test (TDT) in family trios. When analyzing family trios, only heterozygous parents are included, expected to transmit either alleles 50% of the time to their children. Significantly increased/decreased allele transmission above 50% is defined as transmission disequilibrium. A positive TDT test suggests that the gene itself influences susceptibility to the disease or is in tight linkage with the disease-predisposing gene (39, 41). The software (www.hsph.harvard.edu/fbat) was used to analyze the pedigrees using the PBAT test for individual variants and statistical power estimation and HBAT for *MBL2* haplotypes (minimum informative families = 10), first testing the additive model, with follow-up testing of dominant and recessive models.

We compared *MBL2* variant frequencies in PICFLU to a population control cohort of healthy children (12–19 years old), from the National Health and Nutrition Examination Survey (NHANES) (35) who had MBL genotyping available (27). We stratified the comparison by the racial groups reported in NHANES (white non-Hispanic, Black non-Hispanic and white Hispanic). We tested for Hardy Weinberg equilibrium in each subgroup and compared frequencies using the allelic test (using Rx64 3.2.1 software package) (42).

In the severity analysis, we a utilized a whole genome association analysis toolset called PLINK (43) (available at https://pngu.mgh.harvard.edu/~purcell/plink/) that uses Chi-square or Fishers Exact tests to evaluate allele and genotype associations. We evaluated associations between the five individual *MBL2* alleles and severity phenotypes using the allelic test (42). Data were analyzed in the entire genotyped cohort, as well as in the major racial ethnic subgroup (white non-Hispanics) to exclude confounding by population admixture. Severity markers included the continuous PRISM-III admission illness severity score (33) (untransformed and Log transformed) and the dichotomous outcomes of ALI/ARDS, extracorporeal life support (ELS), bacterial co-infection, septic shock, and hospital mortality.

To control for multiple comparisons, we used a Bonferroni corrected *p*-value critical value of 0.0083 (0.05/6) adjusted for 5 *MBL2* variants making 6 haplotypes that were tested. Although this adjustment may not be sufficiently stringent given the number of phenotypes tested, the severity phenotypes were not independent.

A priori, we planned to test for interactions with other reported genetic associations in PICFLU, which was with *IFITM3* rs34481144 and mortality (11). IFITM3 rs12252 was not associated with influenza susceptibility or severity in PICFLU (30). We also recently reported higher mortality in children with influenza-MRSA co-infection with vancomycin monotherapy vs. dual anti-MRSA coverage (31). So we used Gaussian linear modeling to check for interaction with antibiotic therapy and associated alleles using the lm function in the R basic statistical library (Supplemental Table 3). We did not correct for multiple comparisons in these secondary analyses.

## Results

We enrolled and genotyped 420 children with confirmed influenza-related critical illness; 333 (80%) had influenza A infection (41% H1N1_pdm_09, 15% H3N2, 23% other), and 81 (19%) had influenza B infection. Table 1 lists the demographic characteristics and clinical course of the patients, including comorbid conditions and complications. Of the 420 children, 159 (38%) developed ALI/ARDS, 165 (39%) developed septic shock requiring vasopressors and 30 patients died (7%). Influenza-MRSA co-infection was present in 33 of 420 children in the cohort (8%) and was a risk factor for mortality (*p* < 0.0001) present in 37% of fatalities but only 6% of survivors. Influenza-MSSA co-infection was identified in 41 children (10%), but was present approximately equally (~10%) in deaths and survivors (*p* = 0.1)

**Table 1:** Demographic and clinical characteristics, clinical course, and outcomes of the critically ill children in the PICFLU cohort including a comparison of those with fatal vs. non-fatal infection.

	**Total**	**Fatal**	**Survived**	***P*-Value^∧^**
	**(N = 420)**	**(N = 30)**	**(N = 390)**	
**Female, N (%)**	163 (38.8)	14 (46.7)	149 (38.2)	0.4
**Hispanic, N (%)**	113 (26.9)	11 (36.7)	102 (26.2)	0.2
**Race, N (%)**				0.3
White	305 (72.6)	22 (73.3)	283 (72.6)	0.9
African-American	64 (15.2)	2 (6.7)	62 (15.9)	
Asian	14 (3.3)	1 (3.3)	13 (3.3)	
Mixed or Other	37 (8.8)	5 (16.7)	32 (8.2)	
**Age, years, med (IQR)**	6.5 (2.4, 11.2)	10.1 (1.7, 14.2)	6.3 (2.4, 10.9)	0.09
Baseline Health Status^*^, N (%)				
Previously Healthy	226 (53.8)	17 (56.7)	209 (53.6)	0.7
Respiratory^*^	140 (33.3)	4 (13.3)	136 (34.9)	
Immune Compromised^*^	6 (1.4)	3 (10.0)	3 (0.8)	
Other^*^	177 (42.1)	13 (43.3)	164 (42.1)	
**Support and Complications, N (%)**				
Acute Lung Injury	159 (37.9)	23 (76.7)	136 (34.9)	**<0.0001**
Myocarditis	11 (2.6)	3 (10.0)	8 (2.1)	0.04
Encephalitis	10 (2.4)	0 (0)	10 (2.6)	1.0
Shock Receiving Vasopressors	165 (39.3)	138 (35.4)	27 (90.0)	**<0.0001**
Extracorporeal Life Support	46 (11.0)	17 (56.7)	29 (7.4)	**<0.0001**
**PRISM Score, median (IQR)**	5 (2, 11)	26.5 (11, 34)	5 (2, 10)	**<0.0001**
Bacterial Co-infection^**^, N (%)	133 (31.7)	18 (60.0)	115 (29.5)	**0.0005**
Methicillin-resistant *S. aureus*	33 (7.9)	11 (36.7)	22 (5.6)	**<0.0001**
Methicillin-resistant *S. aureus*	41 (9.8)	3 (10.0)	38 (9.7)	1.0
*Streptococcus pneumoniae*	16 (3.8)	1 (3.3)	15 (3.9)	1.0
Other Bacteria	50 (11.9)	3 (10.0)	47 (12.1)	1.0
**Viral Co-infection, N (%)**	94 (22.6)	6 (20.0)	88 (22.6)	0.7
Influenza Type, N^***^ **(%)**				
Influenza A	333 (79.7)	22 (73.3)	311 (80.2)	0.4
H1N1_pdm_09	170 (40.5)	12 (40.0)	158 (40.5)	
H3N2	68 (16.2)	4 (13.3)	64 (16.4)	
Other or no subtype	95 (22.6)	6 (20.0)	89 (22.8)	
Influenza B	81 (19.3)	8 (26.7)	73 (18.7)	0.3
Influenza A and B	2 (0.5)	0 (0)	2 (0.5)	

* Children could have multiple underlying medical conditions and

** *multiple bacterial co-infections*.

*** *4 patients were Influenza positive with subtype unknown*.

∧ *p-values compare Fatal vs. Survived groups. Bolded values are statistically significant*.

The subgroup of 265 patients enrolled prior to May 2014 with available MBL levels had similar demographics and clinical characteristics to the full cohort. The median MBL level was 1,076 ng/ml (interquartile range [IQR] 666–1,894 ng/mL; range 71–7,028). Forty-four patients had levels <500 and 3 patients had levels <100 ng/ml. As shown in Figure 1B, as would be expected wild type (HYA/HYA) diplotypes had the highest levels (median 2,155; IQR 1,698–2,488) and homozygotes for an “O” (B, C, or D) allele had the lowest levels (17, 38). Using multiple linear regression adjusted for sex, race and age, on average, each promoter L variant carried was predicted to decrease MBL levels by 12% (*p* = 0.04), each promoter X by 35% (*p* < 0.001), and each O allele (B, C, or D) by 56% (*p* < 0.001, full regression model shown in Supplemental Table 1).

**Figure 2:**
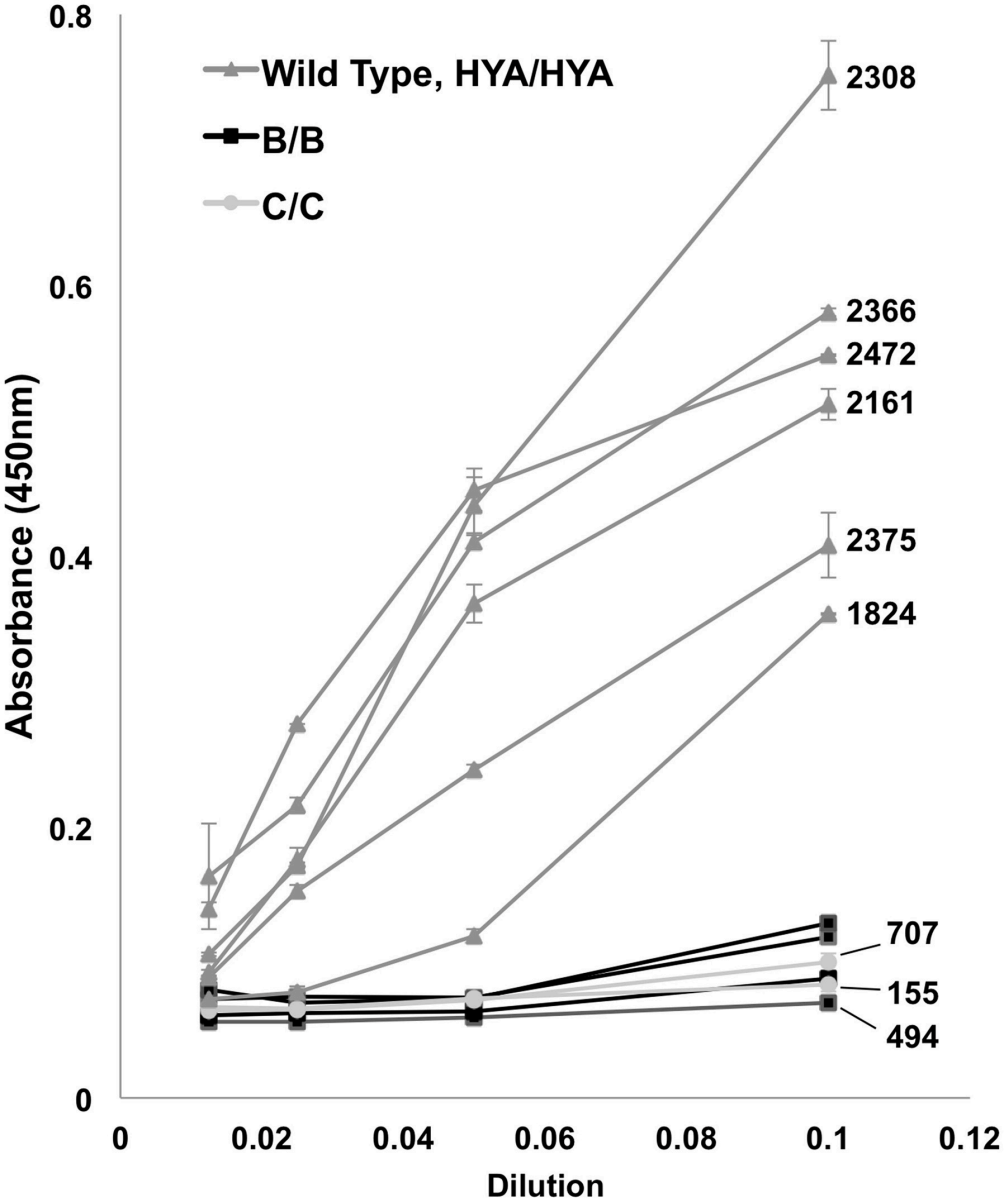
Complement (C3c) fixation by ELISA. Controls (HYA/HYA) fixed complement at typical absorbance (450 nm) measurements despite serial dilutions. Homozygotes [rs1800450_Gly54Asp_(B/B) and rs1800451_Gly57Glu_(C/C)] had markedly reduced complement activation. Bars above and below data points indicate standard deviation. Patients with measured serum levels available have numerical (ng/mL) levels labeled on right. There were no rs5030737_Arg52Cys_(D/D) homozygotes in the population.

The results of the *in vitro* complement fixation analysis are shown in Figure 2. Homozygote B/B or C/C serum had minimal fixation of C3c complement, including those patients with measured serum levels of >500. There were no D/D homozygotes to test. High MBL producing (wild type) controls (HYA/HYA) fixed complement at expected dilutions (17, 36, 37).

Targeted re-sequencing of the *MBL2* exons identified a rare deleterious variant in a white Hispanic patient who had an MBL serum level below the assay's detectable limits (<3.2 ng/mL). This patient was excluded from the gene-to-level analyses. The patient had an HYA/LYA genotype so was predicted to be a high-MBL producer (see Figure 1B). However, he was homozygous for rs74754826 which creates a premature stop codon at aa210 leading to null production of MBL. According to the gnomAD database (gnomAD r2.0.2), the variant is not usually identified in populations of European or Hispanic origin and is overall rare (frequency 0.0006, http://gnomad.broadinstitute.org/variant/10-54528016-C-A) with a relatively higher frequency in populations of African origin (MAF = 0.006). The patient was born prematurely and the respiratory viral culture grew cytomegalovirus at time of influenza diagnosis. The patient had recurrent upper and lower respiratory infections before admission and after discharge. We identified no other individuals with rare, potentially deleterious mutations in *MBL2*.

The results of the individual SNP (PBAT) and haplotype (HBAT) family-based analyses are shown in Table 2. Estimated statistical power in PBAT was acceptable for low MBL-producing variants (X, B, C, and D) but not for the L variant, which is in tight linkage with the other low-producing variants and has minimal influence on MBL levels by itself. From 633 available parents there were 252 nuclear families. Due to high linkage disequilibrium, the five variant alleles combined into six common (≥5% frequency) haplotypes with the X, B, C, and D alleles each represented by one haplotype (17, 38). No transmission disequilibrium was detected for MBL variants or haplotypes (*p* ≥ 0.06 for all analyses).

**Table 2:** Results of the Family Based Association Test Analysis in children with influenza using PBAT for individual SNPs and HBAT for haplotypes.

**SNP (allele)**	**Frequency**	**Families**	***Z*-Score**	***P*-value^∧^**	Power^*^
H (rs11003125)	0.39	166	−1.74	0.08	0.05
L	0.61		+1.74		
Y (rs7096206)	0.84	126	−0.34	0.73	1.00
X	0.16		+0.34		
B (rs1800450)	0.14	104	+1.66	0.10	1.00
Wild Type	0.86		−1.66		
C (rs1800451)	0.05	36	−1.76	0.08	1.00
Wild Type	0.95		+1.76		
D (rs5030737)	0.05	51	−0.14	0.89	1.00
Wild Type	0.95		+0.14		
**Haplotype**	**Frequency**	**Families**	***Z*****-Score**	***P*****-value**^∧^	
HYA	0.34	158	−1.73	0.08	–
LYA	0.25	151	0.75	0.45	–
LXA	0.16	121	0.53	0.60	–
LYB	0.14	97	1.88	0.06	–
LYC	0.05	31	−1.72	0.09	–
HYD	0.05	45	0.17	0.87	–

* *Calculated using the Additive Model in PBAT*.

∧ *p-values are using the Additive Model*.

**Figure 3:**
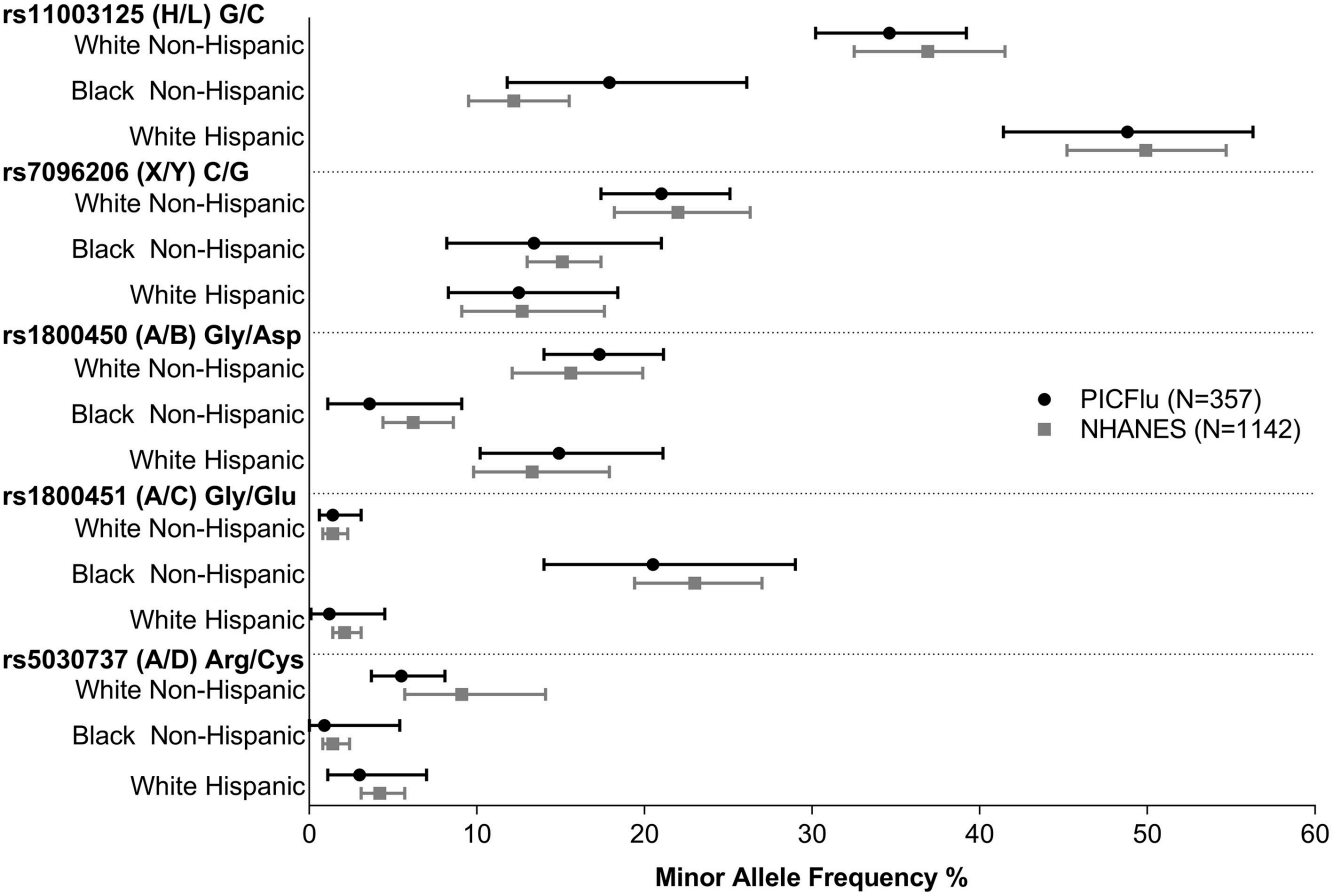
PICFLU MBL variant frequencies, compared with NHANES pediatric population reference, by ethnicity/racial subgroups. Frequencies of all variants were similar (*p* > 0.05 for all comparisons). The calculated 95% confidence interval around the allelic point estimate is shown.

Figure 3 shows the *MBL2* variant frequencies in the PICFLU cohort (for whom self-reported race and ethnicity were available, *n* = 357) compared to the NHANES pediatric population (*n* = 1,142) sub-grouped by race and ethnicity (data provided in Supplemental Table 2 and individual patient data available upon request). No significant differences were identified (all *p* > 0.05). HWE was *p* > 0.05 in all subgroups except the X allele was out of HWE in the white Hispanic NHANES controls.

**Figure 4:**
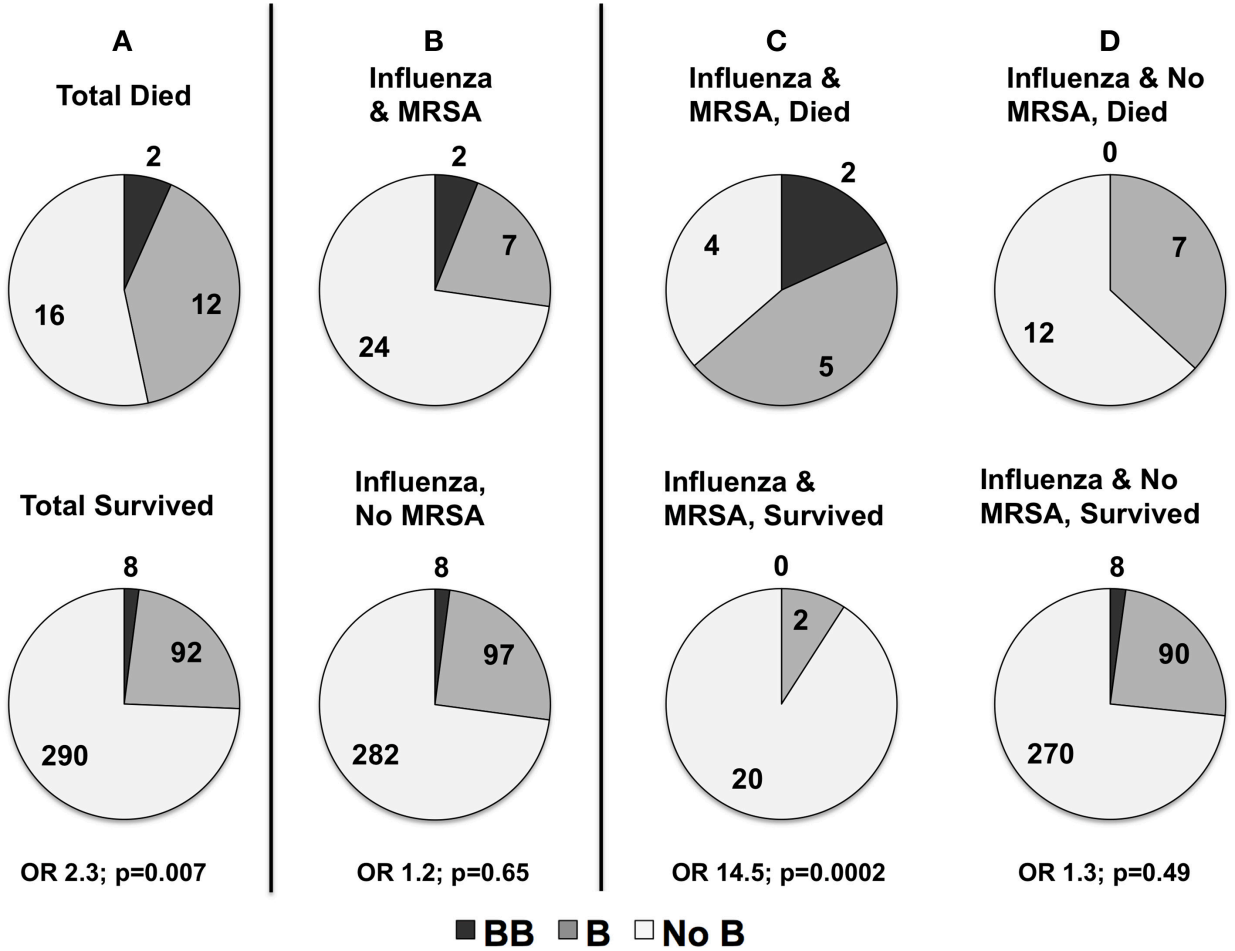
**(A)** Distribution of rs1800450_Gly54Asp_(B) in children with influenza critical illness who died or survived (numbers shown) to hospital discharge in all children (*n* = 420). Mortality was higher in all children carrying at least one copy of the rs1800450_Gly54Asp_(B) allele (*p* = 0.007). **(B)** Distribution of rs1800450_Gly54Asp_(B) in children with and without methicillin-resistant *Staphylococcus aureus* (MRSA) co-infection. There was no association with risk of contracting MRSA co-infection (*p* = 0.65). However, sub-analysis of deaths in patients with MRSA co-infection compared to those without MRSA co-infection showed a strong association of rs1800450_Gly54Asp_(B) with mortality in the patients with MRSA co-infection. **(C)** rs1800450_Gly54Asp_(B) Association with MRSA Co-infection Died vs. MRSA Co-infection Survived (*p* = 0.0002). **(D)** No MRSA Died vs. No MRSA Survived (*p* = 0.49).

In the severity analyses, none of the individual *MBL2* variants were associated with overall illness severity (PRISM III score), or with frequency of shock requiring vasopressors, ALI, or bacterial co-infection (all *p* > 0.05). Carriage of the B missense mutation was higher in children that died. As shown in Figure 4A, in children that died 47% (14/30) carried at least one B mutation compared to 26% (100 of 390) survivors (OR 2.3, *p* = 0.007 overall; white non-Hispanics *n* = 217, OR 2.9, *p* = 0.007). The B mutation was not associated with the frequency of MRSA co-infection (*p* = 0.65, Figure 4B). MRSA co-infection was a risk factor for death, however, and 64% (*n* = 7/11) of children with influenza-MRSA co-infection that died carried at least one B mutation compared to 9% (2/22) survivors with influenza-MRSA co-infection (OR 14.5; *p* = 0.0002; see Figure 4C). In influenza-infected children who died without MRSA co-infection, 37% (*n* = 7/19) carried at least one B mutation compared to 27% (*n* = 98/368) of survivors (OR 1.3, *p* = 0.49, see Figure 4D).

The characteristics and clinical course of the 11 children that died with influenza-MRSA co-infection are shown in Table 3. All children received fluid resuscitation for severe shock and required vasopressors. Ten (91%) were supported via extracorporeal life support (ELS) before death. Although older age was associated with mortality (see Table 1), age was not associated with B allele carriage (*p* = 0.48) so it was not a confounder. The B allele was also not associated with *IFITM3* variant rs34481144 which we previously reported was associated with mortality in PICFLU (11).

**Table 3:** Characteristics and *MBL2* genotype and MBL level (when available) of children with fatal influenza MRSA co-infection in the PICFLU cohort.

**Age group/gender**	**Influenza type**	***MBL2* haplotype**	***MBL2* mutation**	**MBL level**	**Risk factors^∧^**
13–18 yrs/Male	Influenza B	HYA/LYB	rs1800450 (B)	1063^*^	None
13–18 yrs/Female	Influenza A H3	HYA/LYB	rs1800450 (B)	Not available	None
13–18 yrs/Male	Influenza B	LXA/LYB	rs1800450 (B)	666^*^	None
13–18 yrs/Male	Influenza B	LXA/LYB	rs1800450 (B)	570	None
13–18 yrs/Male	Influenza B	LYB/LYC	rs1800450 (B), rs1800451 (C)	338^*^	None
5–12 yrs/Male	Influenza A(H1N1)pdm09	LYB/LYB	rs1800450 (B/B)	1025^*^	None
13–18 yrs/Male	Influenza A(H1N1)pdm09	LYB/LYB	rs1800450 (B/B)	Not available	None
13–18 yrs/Male	Influenza A H3	LYC/LYC	rs1800451 (C/C)	707	None
13–18 yrs/Female	Influenza B	HYA/HYA	none	1417^*^	None
5–12 yrs/Male	Influenza A(H1N1)pdm09	LYA/LYA	none	1042^**^	None
13–18 yrs/Male	Influenza A(H1N1)pdm09	LYA/LYA	none	708^*^	Asthma

*Carriage of one or more copies of the rs1800450(B) allele was present in 7 of the 11 children*.

∧ *All patients were intubated, and met criteria for both shock and ARDS. All patients except one received extracorporeal life support (ELS) prior to death*.

* MBL levels measured after initiation of ELS or

** *unknown when MBL level measured in relationship to ELS initiation*.

We previously published an association between vancomycin monotherapy and death in influenza-MRSA co-infected patients (31), therefore in a secondary analysis we evaluated antibiotic monotherapy as a potential explanation for the association of the B mutation carriage and death. There was no association between carriage of a B mutation and the number (monotherapy vs. two or more) of anti-MRSA antibiotics received in the first 24 h of PICU admission (*p* = 0.44). Multiple logistic regression confirmed an association between anti-MRSA monotherapy and increased mortality (*p* = 0.007) and an independent mortality association with carriage of one B mutation (*p* = 0.0007). There were only two BB homozygotes with MRSA co-infection and both died, precluding comparison.

## Discussion

In North American children and adolescents admitted to the PICU with influenza virus infection during the 2008 to 2016 influenza seasons, *MBL2* variants predicting low MBL levels did not explain disease susceptibility. The four *MBL2* low-producing variants did influence MBL serum levels and the B and C alleles decreased MBL functional activity as previously described (17, 18, 38). Carriage of low-producing *MBL2* variants was not associated with higher disease severity on admission, with development of ALI or shock, or with diagnosis of bacterial co-infection. MBL deficiency does not appear to confer higher risk for severe influenza infection or influenza-related complications.

We found MBL did not increase susceptibility to severe influenza infection in pediatric patients. Overall, the literature on whether low MBL predisposes to severe infections is conflicting. A systematic review and meta-analysis of studies published before 2013 (23) reported increased risk of sepsis susceptibility in children carrying an “O” (B, C, or D) variant and in white adults carrying a B mutation (23). Subsequently, a large study of 1,839 European adults with sepsis from community acquired pneumonia (viral and/or bacterial) or peritonitis showed no association between low-producing *MBL2* variants and infection susceptibility or severity (44). Similarly, a meta-analysis of 5 pediatric studies showed that the B allele was not a risk factor for recurrent respiratory infections in children (45). A meta-analysis of 7 neonatal studies (mostly premature infants) associated low MBL, using levels and predictive variants, with development of sepsis (46). Population-based birth cohort studies showed no increased risk of invasive (bacteremia or meningitis) meningococcal (47) or pneumococcal (48) disease in Danish children carrying low-producing *MBL2* variants. A large cohort of hospitalized children with meningococcemia also did not have higher carriage of O alleles (49). In single center nested case-control PICU studies, we reported no differences in carriage of low-producing *MBL2* variants in patients with severe infections (22) but others reported a 2-fold overall increase in carriage of the O allele with an increase in homozygotes (50). Although *MBL2* haplotypes associated with deficiency appear to be a risk factor for a range of infections in neonates or other immune compromised patients, our study adds to the literature that carriage of low-producing *MBL2* variants does not increase risk of severe viral infections (51). We did identify one child carrying an extremely rare, highly deleterious mutation in *MBL2*, further adding to the literature that rare variants play some role in severe influenza infection susceptibility (8).

We report an association with influenza virus-related mortality and increased carriage of the B mutation in PICFLU, primarily limited to children with MRSA co-infection. The study that stimulated us to evaluate MBL was a pediatric autopsy series reporting of increased carriage of the B mutation in children dying from influenza-MRSA co-infection (27). The autopsy cohort had no survivors for comparison, but 56% (*n* = 5/8) of children dying from influenza-MRSA carried a B mutation (27), significantly higher than the ~14% expected across the population. Similarly, in PICFLU 64% of children dying from influenza-MRSA co-infection carried the B allele (*n* = 7) compared to 9% of children who survived the co-infection. However, the number of influenza-MRSA deaths carrying the B allele across both studies is only 12, precluding any strong conclusions. The association should not be specific to MRSA. Carriage of the B allele was not higher overall in children with influenza-MRSA co-infection. Prior reports of the association of MBL deficiency and death have not been replicated. Eisen et al. combined individual patient data from 4 studies in a meta-analysis, using <500 ng/uL to define deficiency, and reported that deficiency increased the risk of death from *Streptococcus pneumoniae* (52). A subsequent study did not replicate this finding (44). The complement system is redundant, which may lead to contradictory results from evaluation of MBL2 variants across different populations. Contradictory results could also be because increased inflammation from complement activation from high-MBL production could make low-producing MBL2 genotypes protective (53). However, in contrast to reports in adult patients, we saw no evidence of increased lung disease severity (25, 54). or organ dysfunction (55) in children and adolescents with higher MBL. Overall, MBL levels in our population were higher than expected for carriers of an O allele (56), which are usually predicted to be <200 ng/ml (21). This may be secondary to fresh-frozen plasma administration (29) during critical illness or from inflammatory phase elevation.

The PICFLU cohort is a major strength of this study, including children and adolescents with influenza critical illness from multiple centers with rigorous diagnostic testing and phenotyping. The majority of children were previously healthy, including almost all of those that died, increasing the ability to identify genetic influences in severe influenza infection. The inclusion of parental DNA facilitated family-based association testing. Use of sera from the influenza-infected homozygote patients allowed us to verify that variant MBL markedly decreased complement activity. We used a rigorous diagnosis of bacterial co-infection requiring microbiologic confirmation and clinical diagnosis. Although we did not have sufficient sera to demonstrate decreased anti-influenza activity in low-MBL producer variants, this has been shown previously (13). This study was *a priori* designed evaluate the association of MBL and other candidate genes with influenza infection, and we did not have consent to perform whole genome or whole exome sequencing (11, 30). We also did not evaluate other proteins in the lectin pathway of compliment activation (57). Because most PICFLU patients were white and not Hispanic, our findings are generalizable mainly to that group. Unfortunately, we were unable to identify a similar prospectively enrolled cohort of children with influenza-related critical illness for further independent validation of our findings, which is a major limitation.

In summary, we conclude that MBL deficiency is not a risk factor for very severe influenza infection in children and adolescents. Children predicted to have MBL deficiency were not at higher risk of more severe critical illness or development of influenza-associated complications such as ALI or bacterial co-infection. We did confirm a previously reported association of higher carriage of the B allele in children that died from influenza-MRSA co-infection, but our confidence in this finding is low due to the small number of patients. It must be noted, that the majority of critical illness from influenza virus at PICFLU sites is preventable by vaccination (58), which in addition to supportive care and influenza antivirals is currently the most effective way to decrease influenza-related mortality.

## Preliminary Versions of This Work has Been Presented at the Following Meetings

PAS 2016: “Mannose-Binding Lectin and Susceptibility to Pediatric Influenza-Related Critical Illness.” Pediatric Academic Societies Annual Meeting, Baltimore, MD. April 2016. Abstract #750580.

SCCM 2018: “Association of Mannose-Binding Lectin with Influenza Critical Illness in Children.” Society of Critical Care Medicine Annual Meeting, San Antonio, TX. February 2018. Abstract #41.

## Ethics Statement

This study was carried out with approval of the Boston Children's Hospital and all participating site Institutional Review Boards with written informed consent from at least one parent or guardian on behalf of the child, and parents consented for their own participation, in accordance with the Declaration of Helsinki. The protocol was approved by the Institutional Review Boards at each enrolling hospital listed in the acknowledgments.

## Author Contributions

EL, W-KY, MS, YZ, PALISI PICFLU Investigators, and AR made substantial contributions to the conception or design of the study. EL, W-KY, MS, JF, AM, MN, YZ, HS, GM, AnS, LL, SW, MH, NC, AdS, KT, PALISI PICFLU Investigators, and AR made contributions to the acquisition, analysis, and interpretation of the data for this work. EL, W-KY, MS, and AR drafted the work and JF, YZ, HS, GM, SW, MH, and KT revised it critically for important content. EL, W-KY, MS, JF, AM, YZ, HS, GM, AnS, LL, SW, MH, NC, AdS, KT, PM, PALISI PICFLU Investigators, and AR gave final approval of the version to be published. This work represents the findings and conclusions of the authors and does not necessarily represent the official position of the Centers for Disease Control and Prevention or the National Institutes of Health.

### Conflict of Interest Statement

MS holds equity in, and consults to, Opsonix Inc. and SlipChip Corp. The remaining authors declare that the research was conducted in the absence of any commercial or financial relationships that could be construed as a potential conflict of interest.
