# Supplementary material for: Evaluation of Mannose Binding Lectin Gene Variants in Pediatric Influenza Virus-Related Critical Illness
Source: Front Immunol. 2019 May 8;10:1005. doi: 10.3389/fimmu.2019.01005 (PMC6518443; doi:10.3389/fimmu.2019.01005)
Supplement: Supplementary file 1 [file Table_1.DOCX]

**Supplemental Table 1:** Multiple linear regression model adjusting for gender, bacterial coinfection, age, race, and influenza status, for associations between variant alleles and serum levels.

E(log MBL level | XAllele, OAllele, BC, R01, SEX, WHITE, AGE_AD, LAllele) =

β0 + β1*XAllele + β2*OAllele + β21*LAllele+ β3*BC + β4*R01 + β5*SEX + β6*WHITE +

β7* AGE_AD_1-4 + β8* AGE_AD_5-12 + β9* AGE_AD_13-17 + β10* AGE_AD_>18

| **Predictor Covariates** | **Coefficient:**  **β** | **p-value** | **Interpretation** |
| --- | --- | --- | --- |
| XAllele | -.43213 | <0.001 | On average, an additional X allele will decrease the MBL level by **35%** after adjusting for all other covariates in the model. |
| OAllele (“B”, “C”, or “D” mutation) | -0.81776 | < 0.001 | On average, an additional O allele will decrease the MBL level by **56%** after adjusting for all other covariates in the model. |
| LAllele | -0.12994 | 0.0351 | On average, an additional L allele will decrease the MBL level by **12%** after adjusting for all other covariates in the model. |
