# Supplementary material for: Evaluation of Mannose Binding Lectin Gene Variants in Pediatric Influenza Virus-Related Critical Illness
Source: Front Immunol. 2019 May 8;10:1005. doi: 10.3389/fimmu.2019.01005 (PMC6518443; doi:10.3389/fimmu.2019.01005)
Supplement: Supplementary file 2 [file Table_2.DOCX]

**Supplemental Table 2.** MBL variant frequencies by ethnicity and race in the PICFLU and NHANES cohorts.

| **Cohort** | **SNP** | **Race** | **Total N** | **Major Alleles**  **N (%)** | **Heterozy-gotes N (%)** | **Homozy-gotes Minor Allele N (%)** | **HWE P-Value** | **Case-Control P-Value (Allelic Test)** |
| --- | --- | --- | --- | --- | --- | --- | --- | --- |
|  |  |  |  | **No L** | **L** | **LL** |  |  |
| PICFLU | rs11003125 | White N-Hisp | 217 | 27 (12.4) | 96 (44.2) | 94 (43.3) | 0.746 | 0.462 |
| NHANES | rs11003125 | White N-Hisp | 266 | 40 (15.1) | 116 (43.6) | 110 (41.3) | 0.305 | Ref |
| PICFLU | rs11003125 | Black N-Hisp | 56 | 2 (3.6) | 16 (28.6) | 38 (67.9) | 0.845 | 0.091 |
| NHANES | rs11003125 | Black N-Hisp | 483 | 8 (1.6) | 102 (21.1) | 373 (77.3) | 0.737 | Ref |
| PICFLU | rs11003125 | White Hisp | 84 | 20 (23.8) | 42 (50.0) | 22 (26.2) | 0.996 | 0.802 |
| NHANES | rs11003125 | Mex. Amer. | 393 | 103 (26.3) | 186 (47.3) | 104 (26.4) | 0.29 | Ref |
|  |  |  |  | **No X** | **X** | **XX** |  |  |
| PICFLU | rs7096206 | White N-Hisp | 217 | 135 (62.2) | 73 (33.6) | 9 (4.1) | 0.824 | 0.649 |
| NHANES | rs7096206 | White N-Hisp | 266 | 161 (60.7) | 92 (34.7) | 13 (4.7) | 0.975 | Ref |
| PICFLU | rs7096206 | Black N-Hisp | 56 | 42 (75.0) | 13 (23.2) | 1 (1.8) | 0.996 | 0.629 |
| NHANES | rs7096206 | Black N-Hisp | 483 | 347 (71.9) | 126 (26.1) | 10 (2.0) | 0.714 | Ref |
| PICFLU | rs7096206 | White Hisp | 84 | 64 (76.2) | 19 (22.6) | 1 (1.2) | 0.755 | 0.973 |
| NHANES | rs7096206 | Mex. Amer. | 393 | 308 (78.3) | 71 (18.0) | 14 (3.6) | <0.001 | Ref |
|  |  |  |  | **No B** | **B** | **BB** |  |  |
| PICFLU | rs1800450 | White N-Hisp | 217 | 146 (67.3) | 67 (30.9) | 4 (1.8) | 0.239 | 0.483 |
| NHANES | rs1800450 | White N-Hisp | 266 | 193 (72.6) | 63 (23.5) | 10 (3.9) | 0.101 | Ref |
| PICFLU | rs1800450 | Black N-Hisp | 56 | 52 (92.9) | 4 (7.1) | 0 (0) | 0.782 | 0.279 |
| NHANES | rs1800450 | Black N-Hisp | 483 | 427 (88.4) | 53 (11.0) | 3 (0.7) | 0.342 | Ref |
| PICFLU | rs1800450 | White Hisp | 84 | 63 (75.0) | 17 (20.2) | 4 (4.8) | 0.065 | 0.602 |
| NHANES | rs1800450 | Mex. Amer. | 393 | 294 (74.9) | 93 (23.7) | 6 (1.5) | 0.659 | Ref |
|  |  |  |  | **No C** | **C** | **CC** |  |  |
| PICFLU | rs1800451 | White N-Hisp | 217 | 211 (97.2) | 6 (2.8) | 0 (0) | 0.836 | 0.929 |
| NHANES | rs1800451 | White N-Hisp | 266 | 259 (97.3) | 7 (2.7) | 0 (0) | 0.828 | Ref |
| PICFLU | rs1800451 | Black N-Hisp | 56 | 36 (64.3) | 17 (30.4) | 3 (5.4) | 0.601 | 0.559 |
| NHANES | rs1800451 | Black N-Hisp | 483 | 288 (59.6) | 168 (34.8) | 27 (5.6) | 0.702 | Ref |
| PICFLU | rs1800451 | White Hisp | 84 | 82 (97.6) | 2 (2.4) | 0 (0) | 0.912 | 0.413 |
| NHANES | rs1800451 | Mex. Amer. | 393 | 376 (95.7) | 17 (4.3) | 0 (0) | 0.661 | Ref |
|  |  |  |  | **No D** | **D** | **DD** |  |  |
| PICFLU | rs5030737 | White N-Hisp | 217 | 193 (88.9) | 24 (11.1) | 0 (0) | 0.389 | 0.040 |
| NHANES | rs5030737 | White N-Hisp | 266 | 221 (83.1) | 42 (15.7) | 3 (1.2) | 0.533 | Ref |
| PICFLU | rs5030737 | Black N-Hisp | 56 | 55 (98.2) | 1 (1.8) | 0 (0) | 0.462 | 0.634 |
| NHANES | rs5030737 | Black N-Hisp | 483 | 469 (97.2) | 14 (2.8) | 0 (0) | 0.747 | Ref |
| PICFLU | rs5030737 | White Hisp | 84 | 79 (94.0) | 5 (6.0) | 0 (0) | 0.779 | 0.423 |
| NHANES | rs5030737 | Mex. Amer. | 393 | 361 (91.9) | 30 (7.7) | 2 (0.4) | 0.123 | Ref |
| White N-Hisp = White Non-Hispanic; Black N-Hisp = Black Non-Hispanic; White Hisp = White Hispanics; Mex. Amer. = Mex. American | | | | | | | | |
| *L allele is the low-producing MBL allele but is not a minor allele in most White Non-Hispanic populations | | | | | | | | |
