# Supplementary material for: Evaluation of Mannose Binding Lectin Gene Variants in Pediatric Influenza Virus-Related Critical Illness
Source: Front Immunol. 2019 May 8;10:1005. doi: 10.3389/fimmu.2019.01005 (PMC6518443; doi:10.3389/fimmu.2019.01005)
Supplement: Supplementary file 3 [file Table_3.DOCX]

**Supplemental Table 3.** Gaussian linear regression model testing for the independent effect of the number of anti-MRSA antibiotics (1 versus 2 or more) and the number of B alleles (0, 1, 2), and their interactions on mortality in children with influenza-MRSA coinfection in the PICFLU cohort.

lm(formula = outcome ~ mrsa$Antibiotics * as.factor(mrsa$Balleles))

Residuals:
    Min      1Q  Median      3Q     Max
-0.3636  0.0000  0.0000  0.0000  0.6364

| **Coefficients:** | **Estimate** | **Std. Error** | **T value** | **Pr(>\|t\|)** |
| --- | --- | --- | --- | --- |
| (Intercept) | 0.3636 | 0.09258 | 3.928 | 0.0005 |
| 2 or more antibiotics | -0.3636 | 0.1258 | -2.891 | 0.007 |
| 1 B allele | 0.6364 | 0.1656 | 3.843 | 0.0007 |
| 2 B alleles | 0.6364 | 0.3207 | 1.984 | 0.06 |
| Interaction: 2 or more  antibiotics and 1 B allele | -0.6364 | 0.2860 | -2.225 | 0.03 |
| Interaction: 2 or more  antibiotics and 2 B alleles | 0.3636 | 0.4521 | 0.804 | 0.43 |
